# Supplementary material for: Interprofessional Education on the Neurology Clerkship for Physical Therapy and Medical Students
Source: MedEdPORTAL. 2023 May 30;19:11316. doi: 10.15766/mep_2374-8265.11316 (PMC10227187; doi:10.15766/mep_2374-8265.11316)
Supplement: Supplementary file 1 — Facilitator Guide.docxIPE on the Neurology Clerkship.pptxExample Schedule.docxSEIEL Survey.docxNeurological Medical Exam Example.docxPT Neurological Exam Example.docx [file mep_2374-8265.11316-s001.zip › C. Example Schedule.docx]

**Appendix C**

**Inter-Professional Education on the Neurology Clerkship for Physical Therapy (PT) and Medical Students**

**Example Schedule**

**Schedule (detailed description)**

Day before IPE activity:

- Patient list to be sent via email the day before IPE and sent to all participants for review.
- Students should review the PowerPoint (Appendix B) and be prepared to discuss patient(s) in detail. If possible, students should try to chart review and/or see patient(s) prior to the IPE session.

Day of IPE activity:

- Pre-Rounds (1 hour):

1. Participants meet and introductions are made
2. First complete pre-activity survey (*Appendix D*)
3. Discuss roles of each discipline – explain your role in the care of the patient
4. **Verbal Communication**: Do a Verbal presentation of the patient (medical students will present patient as they would on rounds; physical therapy students will present patient from PT perspective)

- Bedside Rounds: Examine patient(s) at bedside (1-2 hours):
  1. Each team member should introduce themselves to the patient at the bedside
  2. Examine the patient
     - Medical Students – each student can demonstrate part of the neurological exam and/or necessary general exam
     - PT student – demonstrate neurological PT exam
  3. Discuss any questions that came up during the exam of the patient(s). Provide feedback to the team members.
  4. If time allows, see the next patient.
- Post Rounds Debriefing (1 hour):

1. **Written Communication:** Review PT notes and medical notes to understand effective communication – (e.g., understand the outline of a typical PT and medical note, ask questions and provide feedback to understand what is being conveyed in the progress notes to facilitate communication via written records in the electronic medical record)
2. Complete post-activity survey (*Appendix D*)

**Schedule (outline)**

| **Session** | **Duration** | **Notes** |
| --- | --- | --- |
| Receive and review patient list via e-mail | 45-60 minutes | - Participants should prepare for the session by reviewing the PowerPoint (Appendix B) and patient(s)’ chart(s) prior to the IPE activity |
| Pre-rounds | 1 hour | - Participants will meet and introduce themselves. - Complete pre-activity survey - **Verbal Communication:** Medical student is encouraged to provide a short verbal presentation of the patient to the PT’s and vice versa |
| Bedside rounds | 1-2 hours | Examining the patient:   - Medical Students – each student can demonstrate part of the neurological exam and/or necessary general exam - PT student/resident – demonstrate neurological PT exam - Discuss any questions that came up during the exam of the patient(s). |
| Post-rounds debriefing | 1 hour | - **Written Communication:** Review PT notes and medical notes to understand effective written communication - Complete post-activity survey |
