# Supplementary material for: Interprofessional Education on the Neurology Clerkship for Physical Therapy and Medical Students
Source: MedEdPORTAL. 2023 May 30;19:11316. doi: 10.15766/mep_2374-8265.11316 (PMC10227187; doi:10.15766/mep_2374-8265.11316)
Supplement: Supplementary file 1 — Facilitator Guide.docxIPE on the Neurology Clerkship.pptxExample Schedule.docxSEIEL Survey.docxNeurological Medical Exam Example.docxPT Neurological Exam Example.docx [file mep_2374-8265.11316-s001.zip › D. SEIEL Survey.docx]

# Appendix D Inter-Professional Education on the Neurology Clerkship for Physical Therapy and Medical Students

# Self-Efficacy for lnterprofessional Experiential Learning

# Adapted from Dalhousie University

Please complete this survey **before and after** the Inter-Professional Education (IPE) activity using the same form.

|  |  | |  | |  | |  | |  | |  | |  | | |  | |  | |  |  | |
| --- | --- | --- | --- | --- | --- | --- | --- | --- | --- | --- | --- | --- | --- | --- | --- | --- | --- | --- | --- | --- | --- | --- |
| 1. Please indicate your role (circle) | **PT resident** | **PT student** | | | **Medical Student** | | | Instructions: Using the following scales, please rate your confidence in your ability to carry out some aspects of your role as a student for interprofessional learning: 1 represents very low confidence in your ability and 10 represents high confidence in your ability. | | | | | | | | | | | | | | |
| 1. Have you participated in IPE activities before? (circle) | **Yes** | **No** | | |  | | |  |  |  |  |  |  |  |  |  |  |  |  |  |  |  |
|  |  | **Low Confidence** | |  | |  | | | |  | | **Some Confidence** | |  |  | |  | |  | | | **Very Confident** |
| 1. Working with other students from a different profession to form a team. | Before | 1 | | 2 | | 3 | | | | 4 | | 5 | | 6 | 7 | | 8 | | 9 | | | 10 |
|  | After | 1 | | 2 | | 3 | | | | 4 | | 5 | | 6 | 7 | | 8 | | 9 | | | 10 |
| 1. Working with other students from different professions to resolve problems in the team. | Before | 1 | | 2 | | 3 | | | | 4 | | 5 | | 6 | 7 | | 8 | | 9 | | | 10 |
|  | After | 1 | | 2 | | 3 | | | | 4 | | 5 | | 6 | 7 | | 8 | | 9 | | | 10 |
| 1. Working with other students from different professions to develop a realistic appropriate patient care plan. | Before | 1 | | 2 | | 3 | | | | 4 | | 5 | | 6 | 7 | | 8 | | 9 | | | 10 |
|  | After | 1 | | 2 | | 3 | | | | 4 | | 5 | | 6 | 7 | | 8 | | 9 | | | 10 |
| 1. Working with other students from different professions to understand our respective roles in an IPE team. | Before | 1 | | 2 | | 3 | | | | 4 | | 5 | | 6 | 7 | | 8 | | 9 | | | 10 |
|  | After | 1 | | 2 | | 3 | | | | 4 | | 5 | | 6 | 7 | | 8 | | 9 | | | 10 |
| 1. Working with other students from different professions to understand the benefits to patients of team care. | Before | 1 | | 2 | | 3 | | | | 4 | | 5 | | 6 | 7 | | 8 | | 9 | | | 10 |
|  | After | 1 | | 2 | | 3 | | | | 4 | | 5 | | 6 | 7 | | 8 | | 9 | | | 10 |
| 1. Understanding and discussing the objectives of interprofessional learning. | Before | 1 | | 2 | | 3 | | | | 4 | | 5 | | 6 | 7 | | 8 | | 9 | | | 10 |
|  | After | 1 | | 2 | | 3 | | | | 4 | | 5 | | 6 | 7 | | 8 | | 9 | | | 10 |
| 1. Interacting with students from other professions and disciplines than my own. | Before | 1 | | 2 | | 3 | | | | 4 | | 5 | | 6 | 7 | | 8 | | 9 | | | 10 |
|  | After | 1 | | 2 | | 3 | | | | 4 | | 5 | | 6 | 7 | | 8 | | 9 | | | 10 |
| 1. Providing feedback to an interprofessional team on our function and work as a team. | Before | 1 | | 2 | | 3 | | | | 4 | | 5 | | 6 | 7 | | 8 | | 9 | | | 10 |
|  | After | 1 | | 2 | | 3 | | | | 4 | | 5 | | 6 | 7 | | 8 | | 9 | | | 10 |
| 1. Providing feedback to individual team members of an IPE team on their function and work on the team. | Before | 1 | | 2 | | 3 | | | | 4 | | 5 | | 6 | 7 | | 8 | | 9 | | | 10 |
|  | After | 1 | | 2 | | 3 | | | | 4 | | 5 | | 6 | 7 | | 8 | | 9 | | | 10 |
| 1. Helping clinical sites understand an interprofessional team's role in a clinical setting. | Before | 1 | | 2 | | 3 | | | | 4 | | 5 | | 6 | 7 | | 8 | | 9 | | | 10 |
|  | After | 1 | | 2 | | 3 | | | | 4 | | 5 | | 6 | 7 | | 8 | | 9 | | | 10 |
| 1. Helping the patient to understand the objectives of the interprofessional learning. | Before | 1 | | 2 | | 3 | | | | 4 | | 5 | | 6 | 7 | | 8 | | 9 | | | 10 |
|  | After | 1 | | 2 | | 3 | | | | 4 | | 5 | | 6 | 7 | | 8 | | 9 | | | 10 |
| 1. Evaluating the quality of the work as an interprofessional team. | Before | 1 | | 2 | | 3 | | | | 4 | | 5 | | 6 | 7 | | 8 | | 9 | | | 10 |
|  | After | 1 | | 2 | | 3 | | | | 4 | | 5 | | 6 | 7 | | 8 | | 9 | | | 10 |
| 1. Evaluating the degree to which an IPE team has achieved its goals. | Before | 1 | | 2 | | 3 | | | | 4 | | 5 | | 6 | 7 | | 8 | | 9 | | | 10 |
|  | After | 1 | | 2 | | 3 | | | | 4 | | 5 | | 6 | 7 | | 8 | | 9 | | | 10 |
| 1. Learning together cooperatively with students from other professions. | Before | 1 | | 2 | | 3 | | | | 4 | | 5 | | 6 | 7 | | 8 | | 9 | | | 10 |
|  | After | 1 | | 2 | | 3 | | | | 4 | | 5 | | 6 | 7 | | 8 | | 9 | | | 10 |
| 1. Communicating effectively with other members of an interprofessional team. | Before | 1 | | 2 | | 3 | | | | 4 | | 5 | | 6 | 7 | | 8 | | 9 | | | 10 |
|  | After | 1 | | 2 | | 3 | | | | 4 | | 5 | | 6 | 7 | | 8 | | 9 | | | 10 |
| 1. Interacting with teachers and preceptors from other professions and disciplines than my own. | Before | 1 | | 2 | | 3 | | | | 4 | | 5 | | 6 | 7 | | 8 | | 9 | | | 10 |
|  | After | 1 | | 2 | | 3 | | | | 4 | | 5 | | 6 | 7 | | 8 | | 9 | | | 10 |

Please identify areas that need improvement for this IPE activity.

|  |
| --- |
|  |
|  |
|  |

Please provide comments on the strengths of this IPE activity.

|  |
| --- |
|  |
|  |
|  |
