# Supplementary material for: Interprofessional Education on the Neurology Clerkship for Physical Therapy and Medical Students
Source: MedEdPORTAL. 2023 May 30;19:11316. doi: 10.15766/mep_2374-8265.11316 (PMC10227187; doi:10.15766/mep_2374-8265.11316)
Supplement: Supplementary file 1 — Facilitator Guide.docxIPE on the Neurology Clerkship.pptxExample Schedule.docxSEIEL Survey.docxNeurological Medical Exam Example.docxPT Neurological Exam Example.docx [file mep_2374-8265.11316-s001.zip › E. Neurological Medical Exam Example.docx]

# **Appendix E**

# **Neurological Medical Exam and Note Example**

# **Handout for Students**

Neurological exam components:

- Vitals – Blood Pressure, Heart Rate, Pulse, Temperature
- Cognition – Is the patient alert, drowsy? Is the patient oriented to name, place, and date?
- Cranial Nerves – Assess:
  - Cranial Nerve 2 – Pupil size and reaction
  - Cranial Nerve 3, 4 and 6 – Extraocular movements
  - Cranial Nerve 5 – Facial sensation in the upper, middle, and lower face bilaterally
  - Cranial Nerve 7 – Facial movement of the upper and lower face
  - Cranial Nerve 8 – Hearing bilaterally
  - Cranial Nerve 9, 10 – Palate raises equally bilaterally, and uvula is midline
  - Cranial Nerve 11 – Sternocleidomastoid and trapezius muscle strength bilaterally
  - Cranial Nerve 12 – Tongue protrudes midline
- Motor – Assess bulk and tone of the muscle, assess strength of isolated muscle groups
- Sensation – Assess light touch and pinprick sensation and assess symmetry in all four extremities
- Reflexes – Assess deep tendon reflexes of the brachioradialis, biceps, triceps, patellar, and ankles bilaterally
- Coordination/Gait – Assess cerebellar function with finger-nose-finger testing and heel-to-shin testing, assess forward walking and note arm swing and stance, assess tandem gait walking

Medical History and Physical written note components:

- Chief Complaint
- History of Present Illness – includes history of events prior to presentation
- Medications
- Allergies
- Past Medical History, Past Surgical History, Family History, Social History
- Exam – including Vitals and Neurological Exam
- Laboratory studies, Diagnostic studies
- Assessment – Summary statement of the patient’s history, relevant exam, relevant lab/imaging findings, and differential diagnoses.
- Treatment Plan – often structured as a list of the patient’s diagnoses and relevant treatment plans/therapies for that diagnosis.
  - Diagnostic studies
  - Laboratory studies
  - Medications
  - Consults
  - Discharge Plans
  - Counseling patient and family
