# Supplementary material for: Interprofessional Education on the Neurology Clerkship for Physical Therapy and Medical Students
Source: MedEdPORTAL. 2023 May 30;19:11316. doi: 10.15766/mep_2374-8265.11316 (PMC10227187; doi:10.15766/mep_2374-8265.11316)
Supplement: Supplementary file 1 — Facilitator Guide.docxIPE on the Neurology Clerkship.pptxExample Schedule.docxSEIEL Survey.docxNeurological Medical Exam Example.docxPT Neurological Exam Example.docx [file mep_2374-8265.11316-s001.zip › F. PT Neurological Exam Example.docx]

**Appendix F**

**Physical Therapy (PT) Neurological Exam and Note Example**

**Handout for Students**

PT considerations prior to beginning a therapy session:

- Precautions: blood pressure stops, helmets, ability to weight bear
- Speak to the Nurse; ask if patient can safely participate in PT
- Social history: determine prior level of function
- Assess patient’s goals of care
- Vitals: assess vitals prior to change in position

Physical Therapy exam:

- Cognition - Is the patient alert, drowsy? Is the patient oriented to name, place, and date?
- Cranial Nerves – Assess:
  - Cranial Nerve 2 – Pupil size and reaction
  - Cranial Nerve 3, 4 and 6 – Extraocular movements
  - Cranial Nerve 5 – Facial sensation in the upper, middle, and lower face bilaterally
  - Cranial Nerve 7 – Facial movement of the upper and lower face
  - Cranial Nerve 8 – Hearing bilaterally
  - Cranial Nerve 9, 10 – Palate raises equally bilaterally, and uvula is midline
  - Cranial Nerve 11 – Sternocleidomastoid and trapezius muscle strength bilaterally
  - Cranial Nerve 12 – Tongue protrudes midline
- Motor – Assess range of motion and tone, assess strength of isolated muscle groups
- Sensation – Assess light touch and pinprick sensation and assess symmetry in all four extremities
- Coordination – Assess cerebellar function with finger-nose-finger testing and heel-to-shin testing
- Sitting balance/Dynamic balance – Assess ability to sit upright without leaning and evaluate posture, assess ability to stand and walk upright without veering or falling to the side and evaluate posture
- Activity Tolerance/Endurance – Measure and evaluate how much activity the patient can tolerate without fatiguing and their potential endurance for therapy
- Function – Assess Levels of Assistance for activity defined as Independent – 0% assistance needed; Minimal Assistance - >25% assistance needed; Moderate Assistance - >50% assistance needed; Maximal Assistance - >75% assistance needed; Dependent - 100% assistance needed
- Outcome Measures: Assess functional physical performance measures, caregiver or self-reported measures, and standardized physical performance battery

Physical Therapy written note components:

- Medical and social/work history
- Social support
- Prior level of function
- Current level of function
- Physical Therapy exam
- Functional Independence Measure – Tool that measures independence for self-care, including sphincter control, transfers, locomotion, communication, and social cognition and determines levels of assistance required for each of these activities
- Assistive Devices – List the assistive devices that the patient requires
- Gait – Describes patient’s level of assistance required in different environments such as the household or neighborhood
- Home Exercise Program – This is a summary statement of the patient’s goals of therapy and exercise plan
- Disposition recommendations and why (examples listed):
  - - Home: Patient must have necessary assistive device, home exercise programs, and be able to independently perform therapy
    - Inpatient rehab: Patient must be able to tolerate 3 hours of therapy
    - Skilled Nursing Facility: Patient requires medical management: infection management, intravenous antibiotic management, etc.
