# Supplementary material for: Interprofessional Education on the Neurology Clerkship for Physical Therapy and Medical Students
Source: MedEdPORTAL. 2023 May 30;19:11316. doi: 10.15766/mep_2374-8265.11316 (PMC10227187; doi:10.15766/mep_2374-8265.11316)
Supplement: Supplementary file 1 — Facilitator Guide.docxIPE on the Neurology Clerkship.pptxExample Schedule.docxSEIEL Survey.docxNeurological Medical Exam Example.docxPT Neurological Exam Example.docx [file mep_2374-8265.11316-s001.zip › A. Facilitator Guide.docx]

**Appendix A**

**Inter-Professional Education on the Neurology Clerkship for Physical Therapy and Medical students**

**Facilitator Guide**

**Inter-Professional Education (IPE) Objectives:**

1. Demonstrate effective communication using tools and techniques to facilitate discussions and interactions that enhance team function

2. Describe how one’s uniqueness contributes to effective communication and positive interprofessional working relationships

3. Explain the roles and responsibilities of other providers and how the team works together to provide care, promote health, and prevent disease.

4. Use unique and complementary abilities of all members of the team to optimize patient care.

5. Report improved self-efficacy when communicating and providing feedback within interprofessional teams.

**Team Participants (ideal participant numbers):**

Total number: 5 participants per patient

1 Facilitator (which can also be the Neurologic Physical Therapy resident), 1-2 Physical Therapy student(s), and 2 Medical Students per patient.

**Overview of Schedule (details in Appendix C):**

Total Time: 4 hours

1. Pre-Rounds (1 hour)
2. Bedside Rounds (1-2 hours)
3. Post-Rounds debriefing (1 hour)

**Patient selection:**

The day prior to the IPE activity, it is suggested that the facilitator identify appropriate patients admitted to the hospital with neurological complaints. The facilitator should select 2-4 potential patients for the IPE activity. More than two patients are often chosen in the event a patient is not available at the time of the IPE activity. These patients should be admitted with a neurological complaint, ideally with a physical neurological deficit (e.g., left sided weakness from stroke, gait difficulties from Parkinson’s disease) and able to communicate and interact with the IPE team. Patients will need to be notified of the activity, when it will occur, and be willing to participate. The facilitator will send out a secure email to all participants with patient names and medical record numbers (MRN) of potential patients that may be seen so that review of the chart can be completed prior to the activity.

*Tip*: It is often helpful if the facilitator can familiarize themselves with the patient’s history and exam prior to the IPE activity. In doing so, the facilitator can focus the students’ attention on portions of the exam that are relevant to the patient’s disease and the patient’s tolerance/endurance for the planned activity.

**Pre-Rounds (1 hour) – Emphasizing Verbal Communication** – Pre-Rounds occur in the team room or can be in the room with the patient:

- Start Pre-rounds with introductions: The participants introduce and identify themselves as physical therapy (PT) students or medical students. Facilitators also introduce themselves.
- Complete pre-activity survey: After introduction, allow students to complete the SEIEL survey (Appendix D).
- Discuss roles of each discipline: Once the survey is completed, ask each student to explain their role in the care of the patient. The facilitator may ask: “Students, would you please explain what your main concern is regarding the patient?” *For example, medical students may respond, “I have been seeing Mrs. X for her left sided weakness and recently we have been addressing her high blood pressures.” The physical therapy student may say, “In the care of Mrs. X, I will be addressing her limited range of motion on the left side.”*
- Verbal Communication: The facilitator asks one student from each specialty to give a brief verbal presentation on the patient you plan to see. The facilitator may ask, “Students, would you please give a short verbal presentation of the patient?” One student from each specialty (medical student and physical therapy student) will present. *For example, the medical student may respond, “Mrs. X is a 56-year-old woman with a history of diabetes and hypertension, presenting 2 days ago with sudden onset of left sided weakness. She has an MRI showing a right sided lacunar stroke. Her blood pressure has been elevated recently and we are working on finding some medications that will help control this better by the time she is discharged.”*
- **The facilitator should now ask both PT and medical students to compare the verbal presentations of each profession.** For example, the facilitator may ask the medical student, “Please point out what differences you see in the therapy goals of care and treatment plans compared to the medical goals of care.”
- *Tip*: Facilitators should emphasize the important medical and/or therapy facts. For example, “It is important to recognize that Mrs. X lives on the second floor and the handrails are on the left side only and thus, her therapy needs and discharge planning will need to take this into consideration.”

**Bedside Rounds (1-2 hours) – Examining the Patient**

- - Go see the patient at bedside and introduce the team members.
  - The facilitator will ask each medical student to demonstrate part of the neurological exam. The facilitator asks, “Medical student A, could you please demonstrate the cranial nerve exam and then afterwards, medical student B, could you please demonstrate the motor exam on the patient?”
  - The facilitator will then ask each medical student, “Why was this part of the exam important in the evaluation of the patient?” *Medical student A may respond, “The cranial nerve exam reveals that the patient has left sided face weakness and helps us localize the stroke to a particular part of the brain involved.”*
  - Next, the facilitator will ask each PT student to demonstrate part of the neurological PT exam. The facilitator may ask, “PT student A, could you please demonstrate the motor exam on the patient and PT student B, could you please then demonstrate the gait exam?”
  - The facilitator will similarly ask each PT student, “Why was this part of the exam important in the evaluation of the patient?” *PT student B may respond, “The gait exam will tell us about the patient’s functional state.”*
- **At this point, the facilitator should emphasize the major differences between the exams of each profession.** The facilitator should ask both PT and medical students, “Could you please point out what interesting similarities and differences you saw in the exam findings from the other profession?” Facilitators should encourage participants to give each other feedback (positive or constructive) about aspects of the bedside rounds. *For example, medical students may realize that consideration of the patient’s physical abilities to tolerate long hours of physical therapy may affect their discharge disposition.*
  - *Tip*: It is important to allow each student to demonstrate a portion of the exam so that active participation is encouraged. Given the limited time however, usually the entire neurological exam cannot be performed. Thus, it is important to focus the students’ attention on parts of the neurological exam that are relevant to the patient’s disease and tolerance/endurance for the IPE activity.
  - *Tip*: The facilitator may also add demonstration of some exam components to the neurologic exam that are important to emphasize. For example, students may perform the motor exam without consideration to testing coordination and balance, which may also affect the patient’s ability to walk.

**Post-Rounds Debriefing (1 hour) – Emphasizing Written Communication** – Post-Rounds Debriefing is best done in the team room with access to the electronic medical record so that written notes can be reviewed.

- Written Communication review: the facilitator will pull up the electronic medical record for the patient.
- Everyone will read through the most recent PT note and medical note.
- The facilitator will ask the medical and PT students how they typically write a note, such as a typical outline of a note. The facilitator may ask, “Students what is important to you when you are writing a progress note about a patient?”
- The facilitator will then ask the medical and PT students what they are trying to convey in the note. The facilitator may ask, “What are you trying to tell other people in this summary statement?”
- Now the facilitator will ask the PT students to review the medical written note and encourage them to ask questions. The facilitator may ask, “PT students what are you looking for in a medical note and what are some areas you do not understand?” *For example, the PT student may not understand some of the abbreviations used.*
- Similarly, the facilitator will ask the medical students to review the PT’s written note and encourage them to ask questions. The facilitator may ask, “Medical students what are you looking for in a physical therapy note and what are some areas you do not understand?” *For example, the medical student may not understand what functional status means.*
- **The facilitator should emphasize the salient differences between the professions’ notes.** The facilitator may ask both PT and medical students, “Could you please point out the similarities and differences in the written notes from the other profession?” *For example, PT students may say that they find that discharge planning is a portion of the written note that is important to highlight.*
- *Tip:* The facilitator may also point out important parts of the notes. The facilitator may want to emphasize that the medical notes regarding how well the blood pressure is controlled is a relevant detail to keep accurate and up to date so that when PT’s review the note, they know if it is safe to interact with the patient.
- *Tip*: Often students will ask about medical facts so that they have a better understanding of the care of the patient, and this is often when the conversation can become sidetracked about medical and therapy aspects of the patient. It is important that the facilitator redirects the conversation to emphasize that **facilitating communication between professions** is the main purpose of the IPE activity.
- At the end of the session, students will complete the post-activity evaluation (Appendix D).
