# Supplementary material for: Interprofessional Education on the Neurology Clerkship for Physical Therapy and Medical Students
Source: MedEdPORTAL. 2023 May 30;19:11316. doi: 10.15766/mep_2374-8265.11316 (PMC10227187; doi:10.15766/mep_2374-8265.11316)
Supplement: Supplementary file 1 — Facilitator Guide.docxIPE on the Neurology Clerkship.pptxExample Schedule.docxSEIEL Survey.docxNeurological Medical Exam Example.docxPT Neurological Exam Example.docx [file mep_2374-8265.11316-s001.zip › B. IPE on the Neurology Clerkship.pptx]

## Slide 1
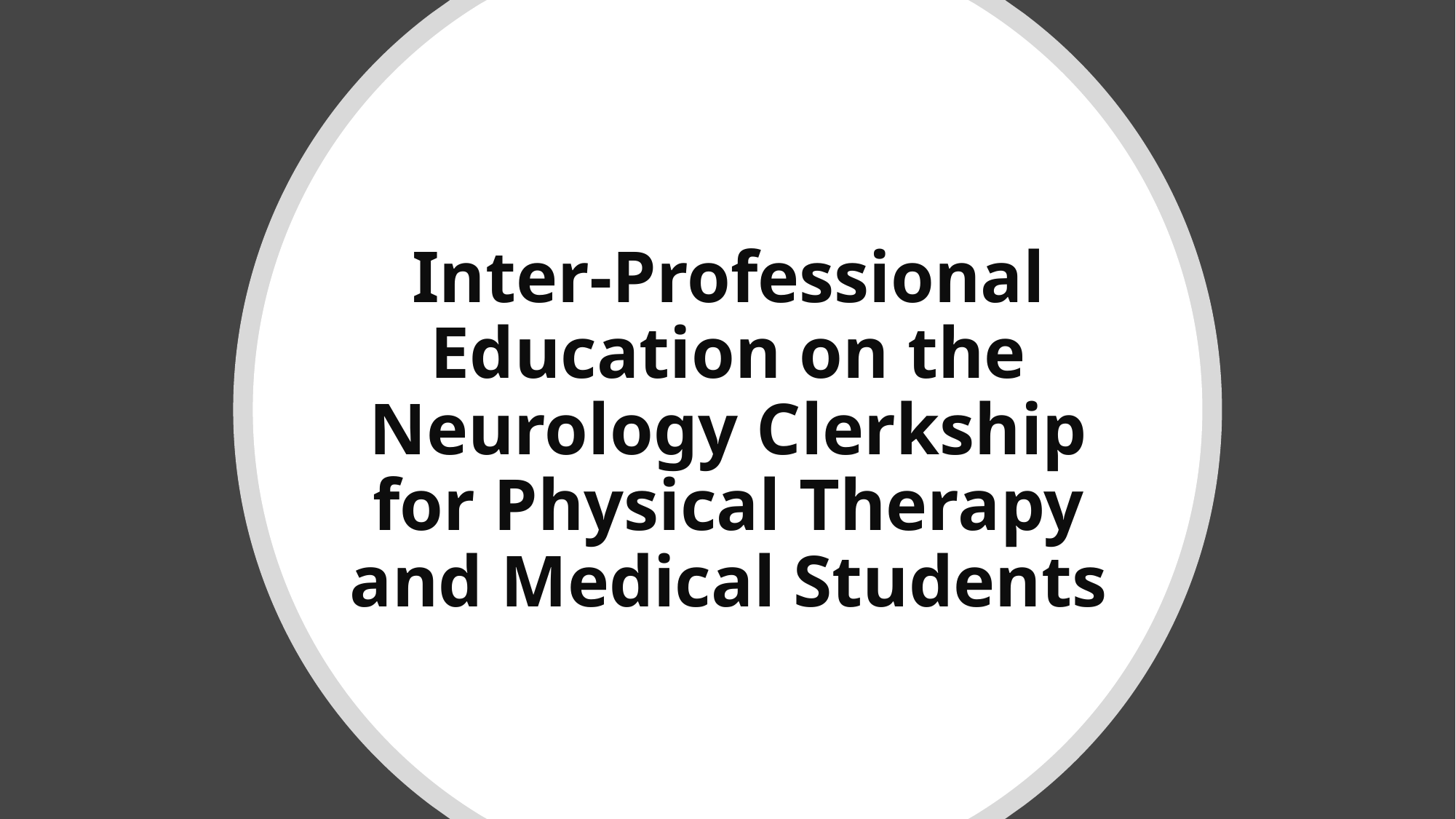

# Inter-Professional Education on the Neurology Clerkship for Physical Therapy and Medical Students

## Slide 2
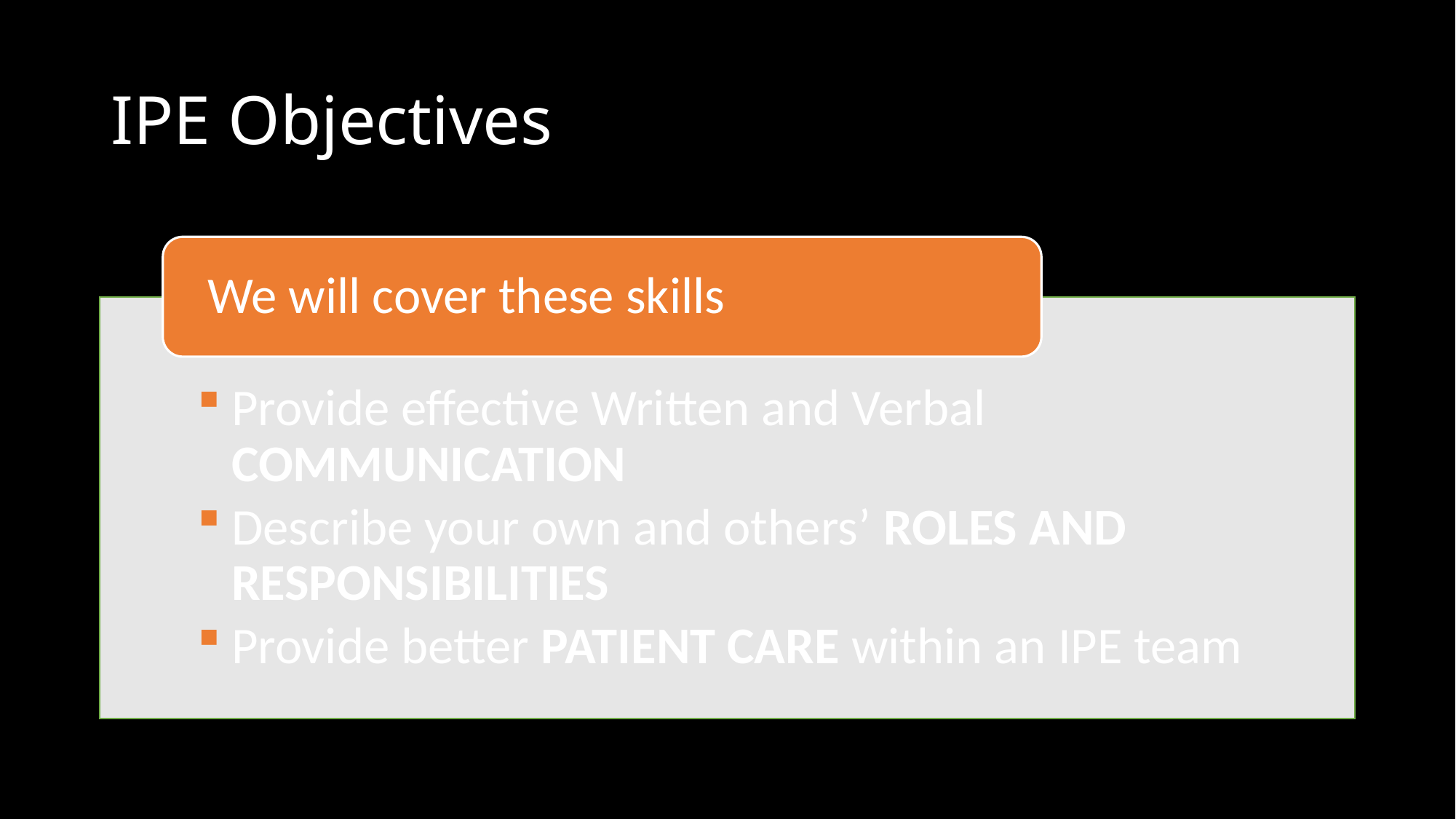

# IPE Objectives

## Slide 3
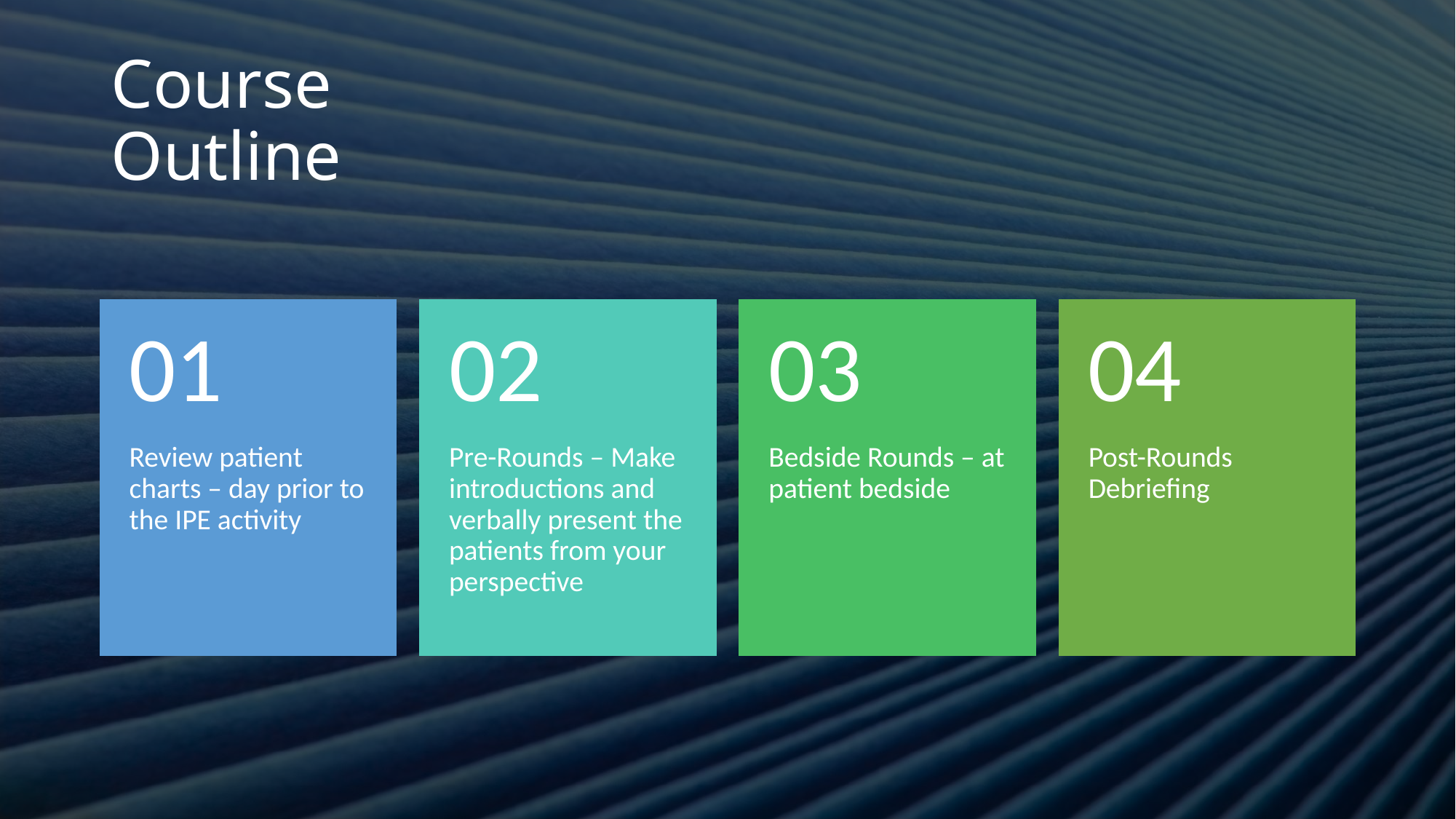

# Course Outline

## Slide 4
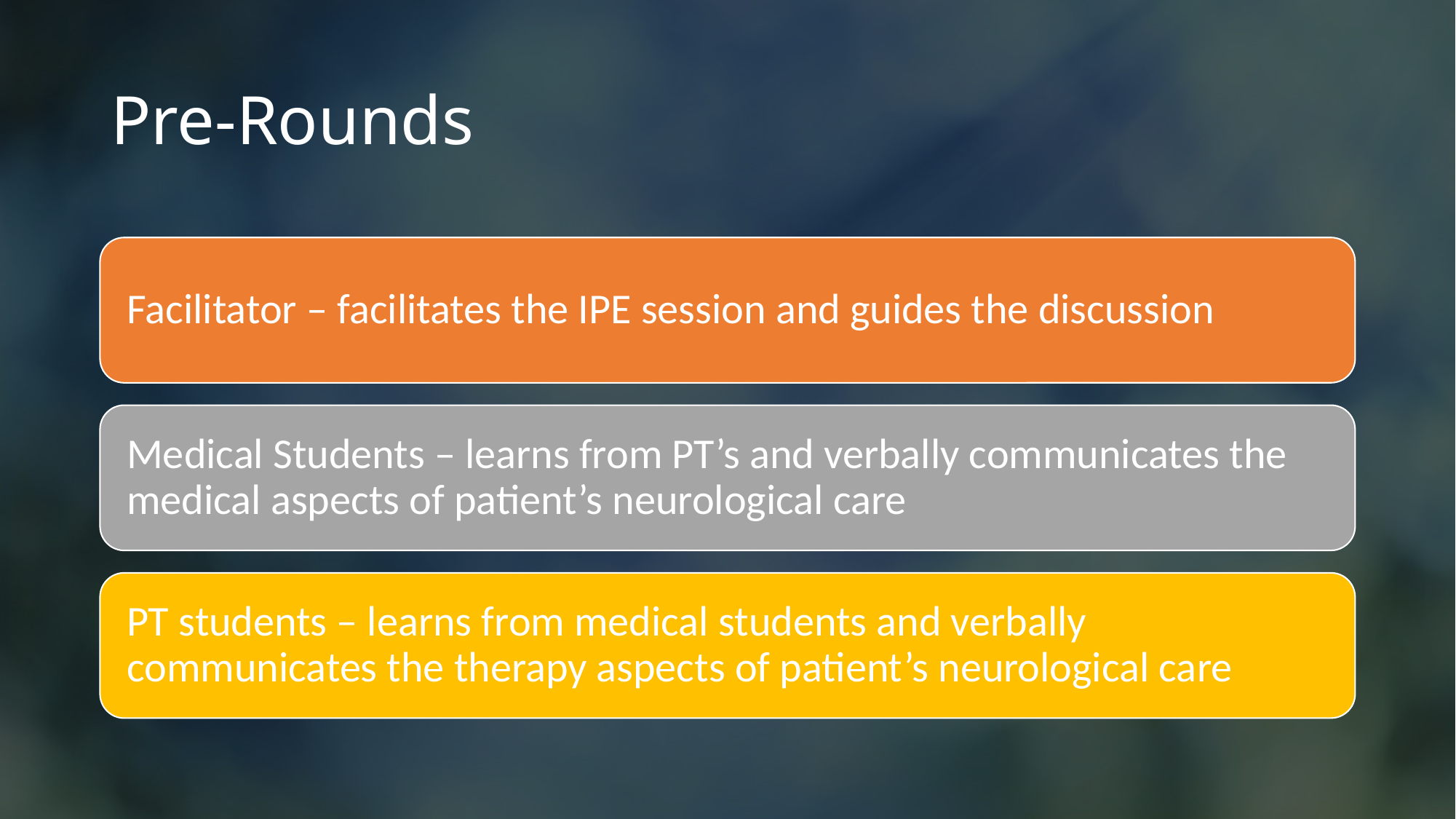

# Pre-Rounds

## Slide 5
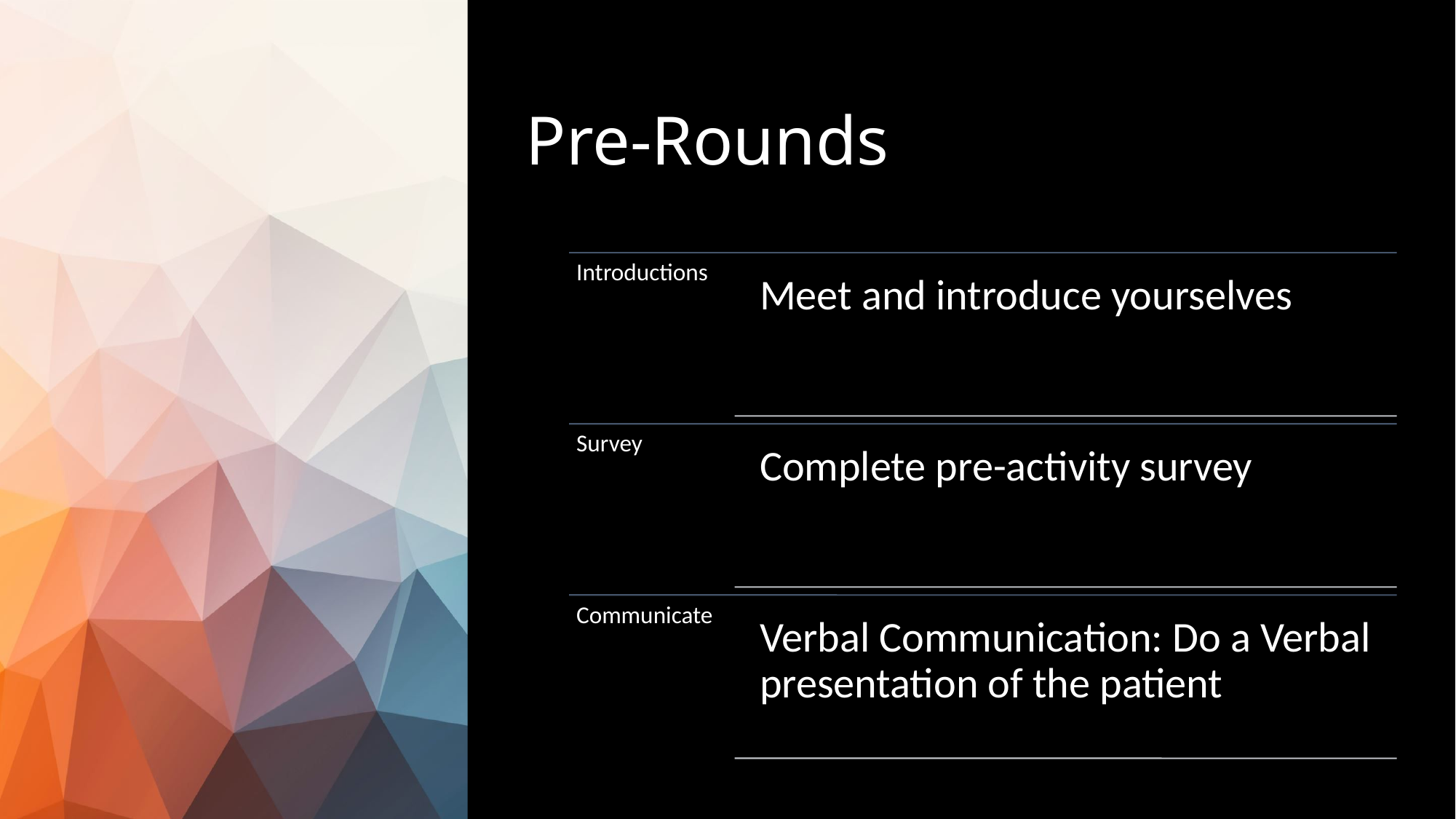

# Pre-Rounds

## Slide 6
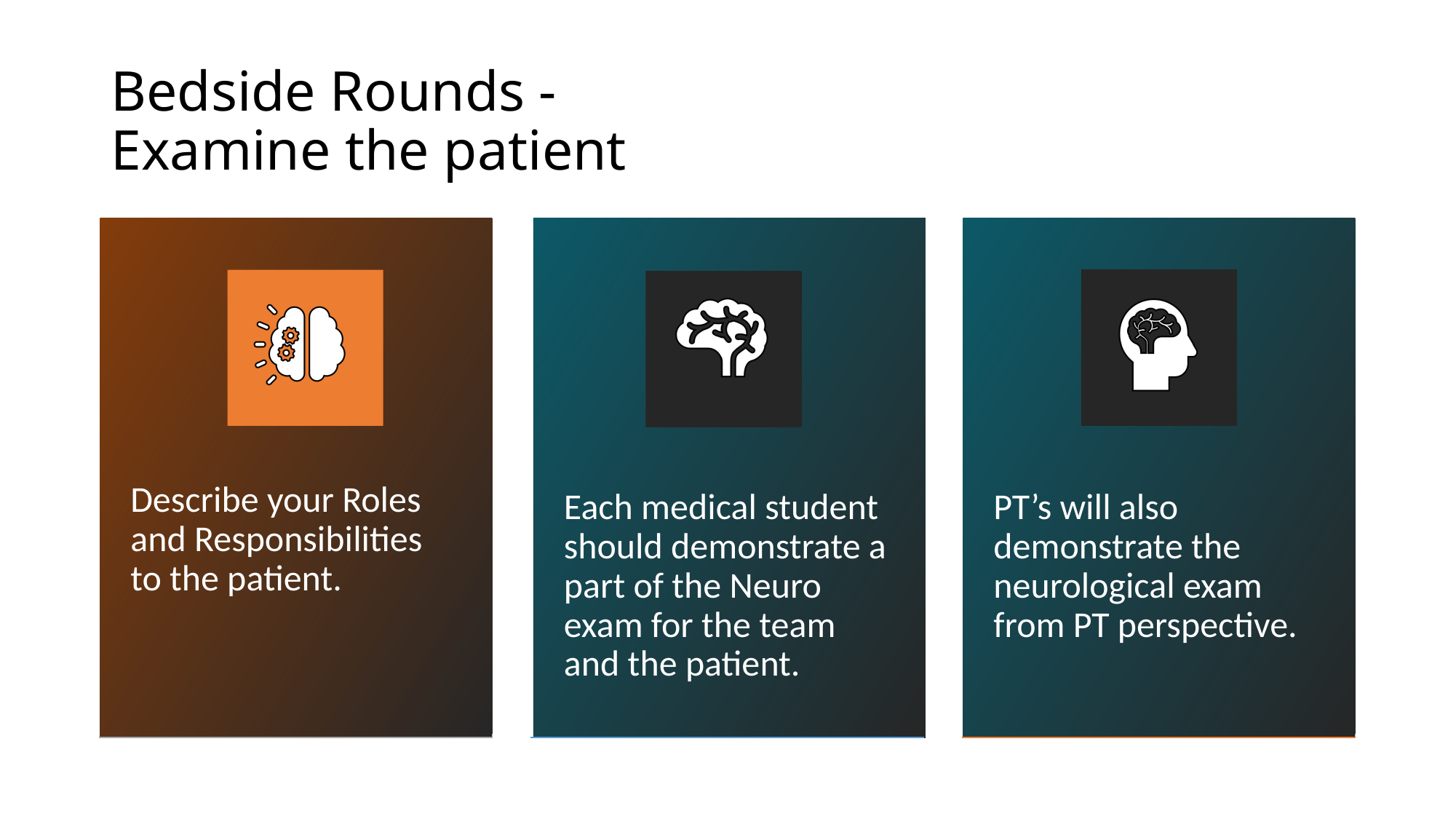

# Bedside Rounds - Examine the patient

## Slide 7
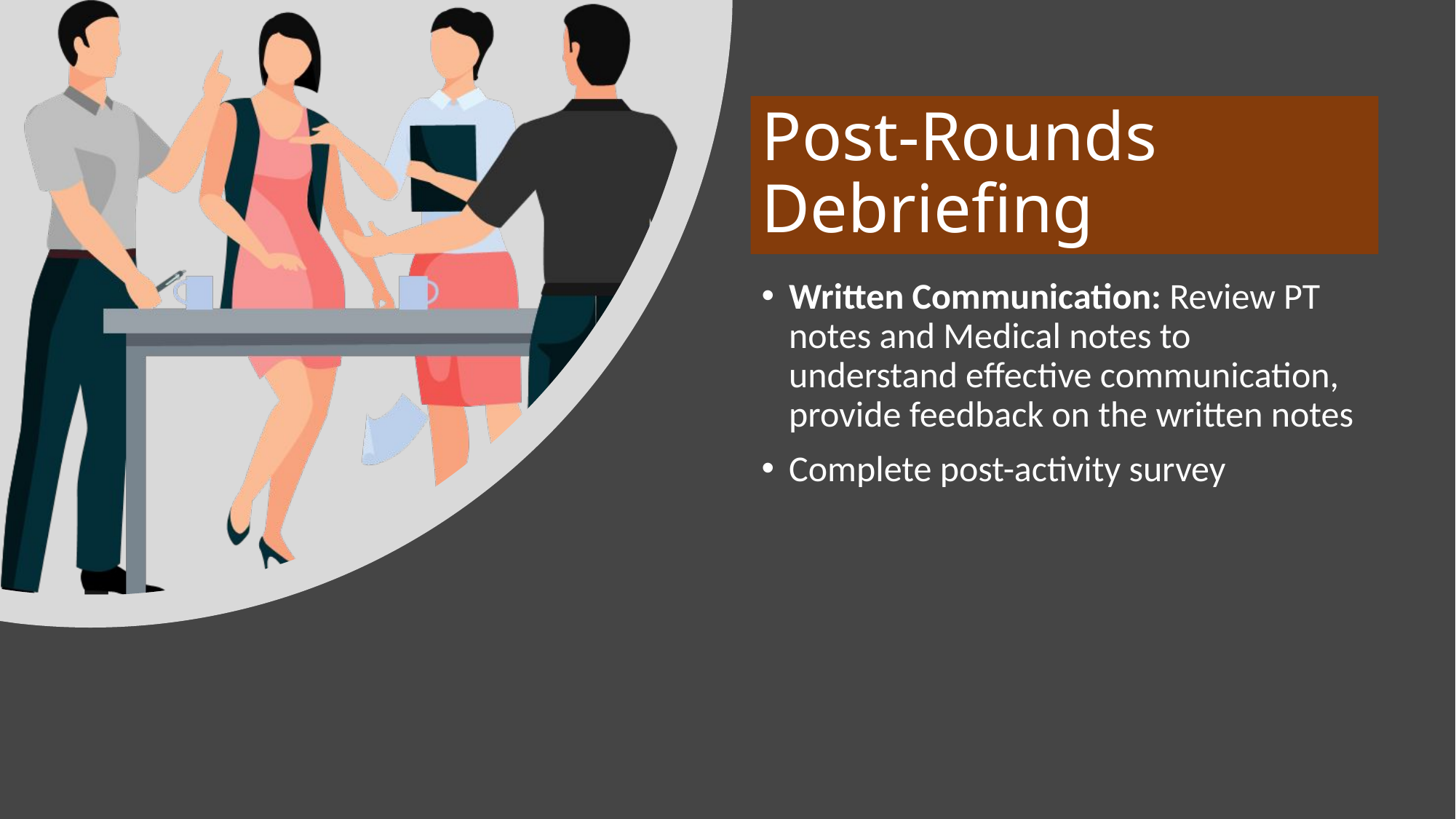

# Post-Rounds Debriefing
Written Communication: Review PT notes and Medical notes to understand effective communication, provide feedback on the written notes
Complete post-activity survey

## Slide 8
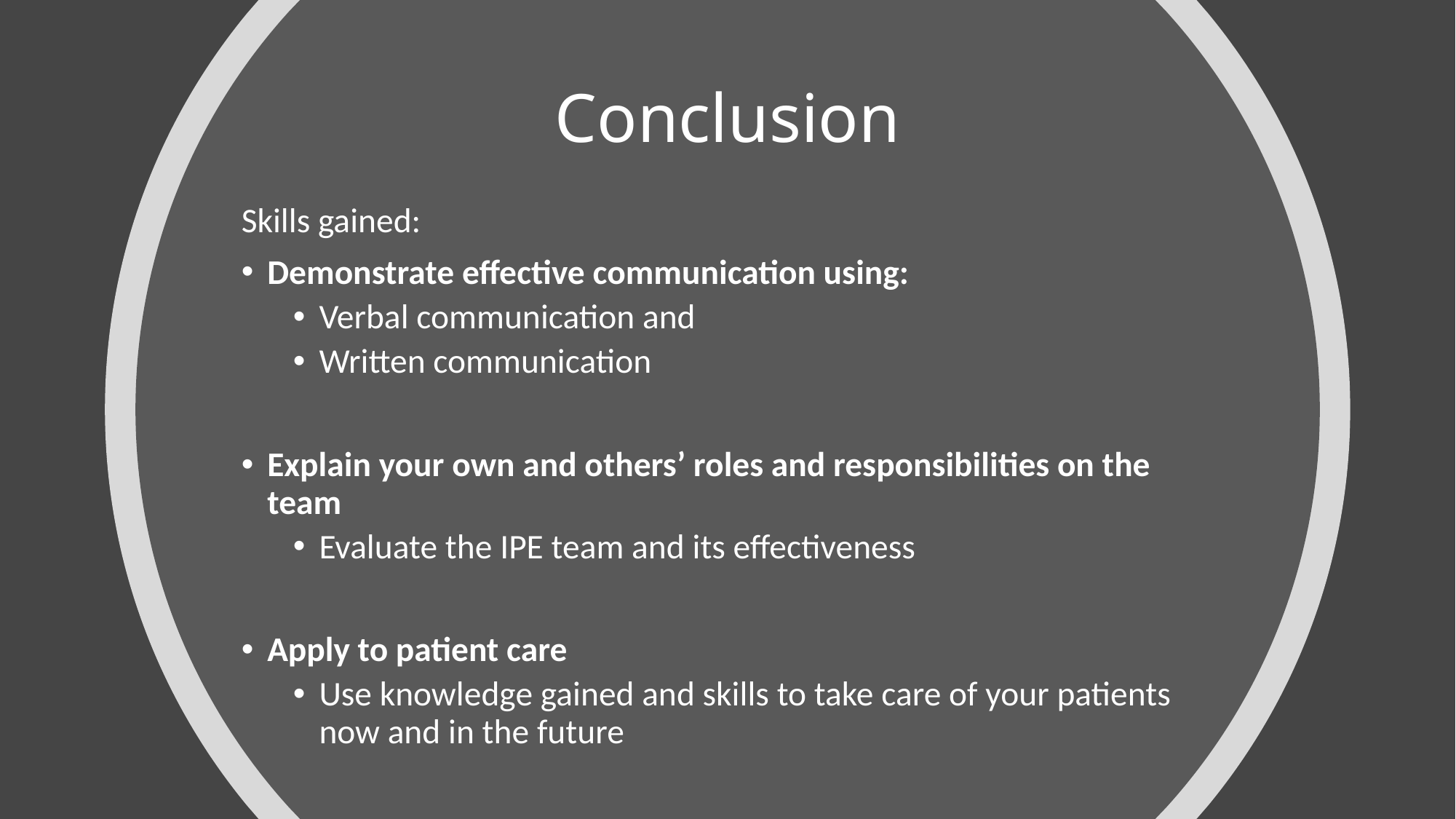

# Conclusion
Skills gained:
Demonstrate effective communication using:
Verbal communication and
Written communication
Explain your own and others’ roles and responsibilities on the team
Evaluate the IPE team and its effectiveness
Apply to patient care
Use knowledge gained and skills to take care of your patients now and in the future
