# Supplementary material for: Interprofessional Education on the Neurology Clerkship for Physical Therapy and Medical Students
Source: MedEdPORTAL. 2023 May 30;19:11316. doi: 10.15766/mep_2374-8265.11316 (PMC10227187; doi:10.15766/mep_2374-8265.11316)
Supplement: Supplementary file 1 — Facilitator Guide.docxIPE on the Neurology Clerkship.pptxExample Schedule.docxSEIEL Survey.docxNeurological Medical Exam Example.docxPT Neurological Exam Example.docx [file mep_2374-8265.11316-s001.zip › Educational Summary Report_11316.pdf]

MedEdPORTAL® | The AAMC Journal of Teaching and Learning Resources

Original Publication Open Access

Interprofessional Education on the Neurology Clerkship for Physical Therapy and Medical Students

Doris Kung, DO*, Wayne Brewer, PT, PhD, MPH†, Victor Oyelami, DPT†, Stephanie Hessel, DPT†, Laurene Bramlett, DPT†, Anne Gill, DrPH†

*Corresponding author: kung@bcm.edu
†Co-second author

Abstract

Introduction: Globally, neurological disorders make up the second most common cause of death and are the leading cause of years lived with disability. Because neurological patients often require multidisciplinary care and future professionals will encounter increasing demands for neurological care, it is important to emphasize education on the interaction between physical therapy (PT) and neurology. Yet there is a dearth of interprofessional education (IPE) learning activities that include neurology clerkship students and physical therapists. **Methods:** We created a 4-hour IPE experience that incorporated hospitalized patients with neurological disorders who were examined at the bedside by pairs of second- and third-year PT students and second- and third-year medical students, followed by a debriefing. Participants completed the Self-Efficacy for Interprofessional Experiential Learning (SEIEL) survey before and after the session. **Results:** Significant pre/post improvements were seen for SEIEL total and domain scores ($n = 75$, $p < .001$). Qualitative comments were analyzed; major themes that emerged included a greater appreciation for the other discipline. Students felt the IPE activity was a great learning opportunity to understand roles and responsibilities and communicate with the other discipline. **Discussion:** Students noted significant increases in their ability to understand and explain the importance of interprofessional communication and in their capabilities as health care professionals to work together on an interprofessional collaborative team. This clinical IPE experience can be seamlessly incorporated into the workplace for medical and PT students. IPE activities like this should be encouraged and developed to reach more students and other health care providers.

Keywords:
Clerkship, Clinical Teaching/Bedside Teaching, Interprofessional Education, Neurology, Physical Therapy, Self-Efficacy

Educational Objectives

By the end of this activity, learners will be able to:

1. Demonstrate effective communication using tools and techniques to facilitate discussions and interactions that enhance team function.
2. Describe how one's uniqueness contributes to effective communication and positive interprofessional working relationships.
3. Explain the roles and responsibilities of other providers and how the team works together to provide care, promote health, and prevent disease.
4. Use unique and complementary abilities of all members of the team to optimize patient care.
5. Report improved self-efficacy when communicating and providing feedback within interprofessional teams.

Citation:
Kung D, Brewer W, Oyelami V, Hessel S, Bramlett L, Gill A. Interprofessional education on the neurology clerkship for physical therapy and medical students. *MedEdPORTAL*. 2023;19:11316.
https://doi.org/10.15766/mep_2374-8265.11316

Introduction

In 2016, the Global Burden of Disease Study showed that neurological disorders such as stroke, traumatic brain injury, spinal cord injury, and Alzheimer's dementia were the number one causes of disability and the second most common causes of death worldwide.[1] With an aging population, the demand for neurological health care, including the need for medical and rehabilitation services, will only increase. Neurology is a discipline that requires precision communication and teamwork to care for medically complex and difficult patients. As such, interprofessional teamwork is essential to providing quality patient care and services. Previous studies have shown improved practice and patient outcomes with neurological diseases when

Copyright © 2023 Kung et al. This is an open-access publication distributed under the terms of the Creative Commons Attribution license.

interprofessional activities are incorporated for various disciplines including physical therapists and neurologists.[2,3] The overarching goals of interprofessional education (IPE) are to improve patient safety for all patients through a better understanding of discipline-specific roles and responsibilities, recognizing how each specialty brings expertise to effective patient care; to improve communication between professions; and to enhance teamwork as described by the Interprofessional Education Collaborative for competent interprofessional collaboration.[4] We recognize that physical therapy (PT) is an essential discipline requiring close collaboration among health care workers and that the neurology clerkship is an ideal venue for teaching these interprofessional skills. Because neurology encompasses both medical and surgical diagnoses, almost all neurology patients admitted to a hospital can benefit from PT. In addition, many disorders require the expertise of and collaboration with physical therapists to ensure a patient's recovery from a variety of neurological diseases. However, as a limited resource, physicians must know when and how to engage physical therapists to make decisions on short- and long-term therapeutic goals. To better gauge what medical students knew about PT, we conducted several focus groups with medical students after they attended classroom PT training at a local school of PT. We discovered that medical students' knowledge of basic PT terminology and therapeutics was grossly deficient and determined that in-person clinical training would provide more efficient and effective IPE knowledge and skills.

Developing new clerkship curricula is complex. The Liaison Committee on Medical Education requires IPE activities in clinical courses and tasks course directors with implementing these curricula to meet the standard.[5] All educational activities must compete for valuable teaching time in an already compressed teaching schedule. Faculty must ensure that an IPE activity is sustainable and that students feel the learning experience provides added value. Student and PT schedules, logistical conflicts, and IPE curricular content are all considerations that must be thoughtfully implemented to make our IPE activity an integrated part of the clerkship experience (Figure).[6]

In creating our IPE experience, we investigated other model curricula.[7-10] We searched *MedEdPORTAL* and discovered several IPE offerings that included physical therapists as possible but not primary participants. One offering included physical therapists in a multidisciplinary simulation for a stroke patient.[7] While intriguing, this offering was geared towards learners in a simulated emergency care environment. A similar offering for medical students and other disciplines was designed to simulate poststroke care.[8] One of our aims was for students to systematically focus on one discipline at a time to enhance their knowledge and promote communication between the two disciplines. Another aim was to provide an integrated workplace experience relevant to the medical students' current experiences on the neurology clerkship and to the PT students'

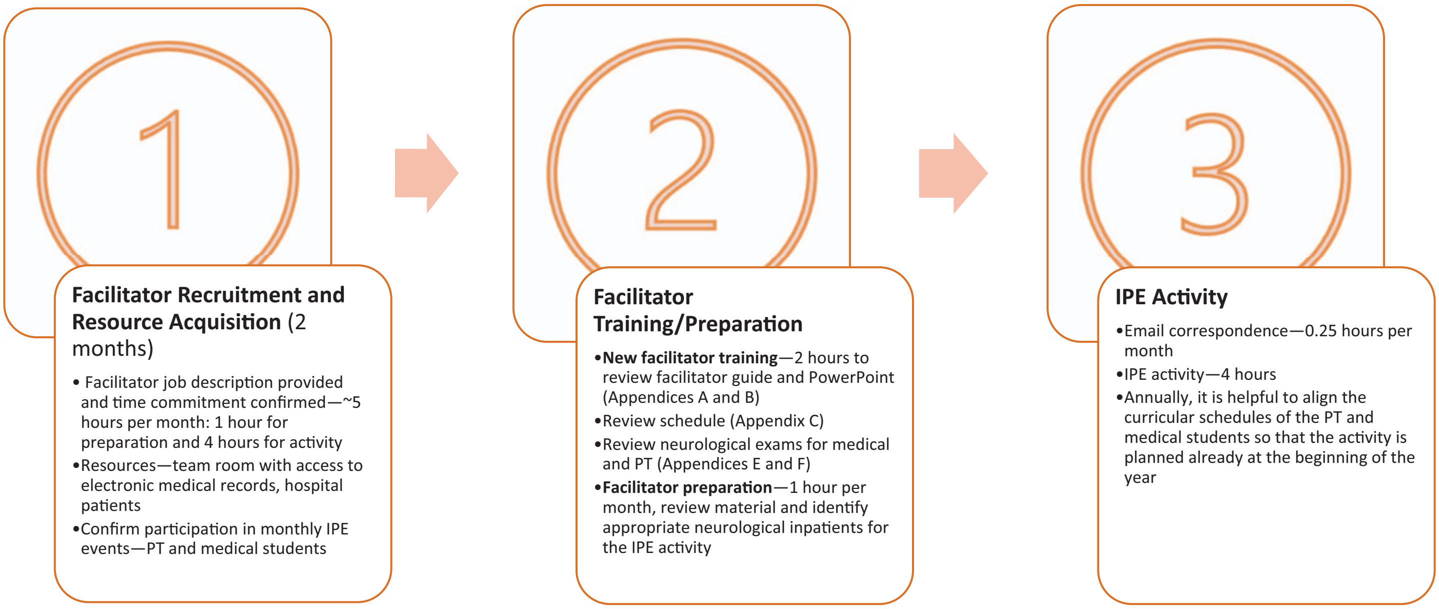

Figure. IPE on the neurology clerkship with PT and medical students—timeline. Abbreviations: IPE, interprofessional education; PT, physical therapy.

Copyright © 2023 Kung et al. This is an open-access publication distributed under the terms of the Creative Commons Attribution license.

curriculum, while incorporating neurology inpatients admitted to the hospital.[6]

Although simulation is important, it has limitations to its authenticity, and direct feedback from the patient's perspective may be lacking. In particular, neurological deficits in patients are difficult to simulate. Simulation is also limited to a focus on one aspect of patient care, whereas, in our IPE activity, there was an opportunity to consider the patient in totality. As much as simulation provides fidelity, hospitalized patients offer an authentic experience for which patient care, therapy, and medical management must be accounted. Our IPE activity employed clinical training, a foundational teaching strategy for all learners in the health care professions.[11] Moreover, we recognized that not only was there a lack of integrated workplace IPE experiences in the medical school but there was also a need for such IPE experiences at the PT schools and residencies.

Social cognitive theory explains that human behavior is influenced by the environment, including the community, personal factors such as knowledge, and behavioral factors such as skills. We used explanative factors, particularly self-efficacy, defined by Bandura as an individual's confidence in their capacity to execute behaviors necessary to produce specific performance attainments, to evaluate students' capabilities in interprofessional collaboration.[12,13] According to Bandura, self-efficacy is the "foundation of [a person's] motivations and accomplishments."[13] Self-efficacy is influenced by several factors, including one's own mastery of and capability in a subject, one's observations in social contexts where comparisons to others are made, persuasion from others, and one's understanding of one's own emotional and physical state to manage and achieve goals.[14] Self-efficacy is an important mediator in leading to behavioral changes. Evaluation of students' perceived self-efficacy in interprofessional settings was absolutely fitting for our purposes since our IPE activity encouraged students to demonstrate their own knowledge and observe other professionals while simultaneously providing continuous real-time feedback and debriefing opportunities. Previous studies of educational interventions designed to change the practice behavior of students in the health sciences have been rooted in self-efficacy, and thus, further exploration of this construct based on previous evidence was deemed appropriate for our IPE experience.[15-19] One of the aims of this IPE activity was to examine if there was an improvement in self-efficacy. We used a 16-question survey created and validated by educators from Dalhousie University in Halifax, Nova Scotia, Canada—the Self-Efficacy for Interprofessional Experiential Learning (SEIEL) survey.[20] The educational intervention and evaluation were approved by Baylor College of Medicine's Institutional Review Board (IRB) and exempted by the hospital (Harris Health) system's IRB.

Methods

Participants

Our IPE activity was a single 4-hour session conducted monthly at Ben Taub General Hospital, a Level 1 trauma center in the Texas Medical Center. Second- and third-year PT students and second- and third-year medical students on the neurology clerkship collaborated on chart reviews, performance of examinations, and discussion of care plans for patients admitted with neurologic disorders**.** A typical group for each month consisted of two PT students, two medical students, and one facilitator. Knowledge about IPE was not required prior to the activity. The medical students had previous exposure to IPE activities in their first and second years of medical school via prior simulated IPE experiences. PT students also had prior IPE exposure with nursing students in their training. However, neither profession had prior experience of IPE activities with the other profession, and so, this IPE activity was unique for the PT and medical students due to its exposure to actual hospitalized patients admitted with primary neurological disorders. Each facilitator was oriented to the activity using a train-the-trainer model. First, the new facilitator would observe another experienced facilitator or faculty conduct the IPE activity and participate in an orientation. The facilitator trainee would then run the next IPE activity under supervision. Lastly, the facilitator trainee became the primary facilitator of the activity. We were fortunate to have PT residents with neurology expertise to facilitate the IPE activities. Having neurologic PT residents facilitate the activity is valuable, although we feel that a physical therapist, neurology resident, or faculty member knowledgeable in the care of neurologic patients would also be appropriate. (For the facilitator guide, refer to Appendix A.)

Activity

Each student participated in one 4-hour IPE session. Prior to the IPE activity, an orientation was provided via a PowerPoint presentation (Appendix B) that explained our objectives and expectations. Detailed instructions regarding the place, schedule of the activity (Appendix C), and survey forms (Appendix D) were sent via email. Example outlines of a typical assessment from a medical student and a physical therapist were also included as a reference for students (Appendices E and F, respectively). The day prior to the activity, the names of the patients to be examined were sent to the students so that chart reviews or routine patient care could be performed. The facilitator chose appropriate patients who had been admitted for a neurologic

Copyright © 2023 Kung et al. This is an open-access publication distributed under the terms of the Creative Commons Attribution license.

diagnosis, preferably with those with a physical neurological deficit, with the ability to communicate, and who were cognitively and physically able and willing to participate in the IPE activity.

The IPE team was typically made up of one neurologic PT resident (facilitator), one to two PT students, and one to two medical students. In the first 60 minutes of the activity, called Pre-Rounds, the group was first asked to complete the preactivity evaluation (Appendix D). Following this, students were encouraged to communicate their discipline-specific roles and responsibilities in the care of the patient and to verbally present the patient to each other with medical and therapy focus. Students were encouraged to point out the similarities and differences in the presentations by each specialty. Following the Pre-Rounds session, the team went to the patient's bedside to perform a neurological examination under the supervision of the facilitator. During Bedside Rounds, each student member of the IPE team performed a neurological exam based on their clinical background while the other members of the team observed. The patient examination typically took 1 hour to complete per patient; if time permitted, another patient was examined and discussed. Students were encouraged to point out the similarities and differences in the exams conducted by each specialty. The team then held a debriefing session, called Post-Rounds Debriefing, to discuss the patient(s), review written notes from the medical students and physical therapists, explain discipline-specific terminology or jargon in the patient chart(s), and make recommendations for patient care. During Post-Rounds Debriefing, students discussed written notes from the other profession. This was a time for participants to examine the written notes for their effectiveness in conveying necessary information to the other profession. Students were encouraged to point out the similarities and differences in the written notes by each specialty. Feedback could be given regarding any communication, verbal or written, so that team members could understand the goals for patient care. The facilitator was also encouraged to provide feedback to participants throughout the IPE activity. The same evaluation (Appendix D) was then completed postactivity. The Post-Rounds Debriefing session took approximately 60 minutes to complete.

Outcome Measures

Students completed the SEIEL (Appendix D) before and after the IPE session, and the data presented are from the months of January 2021 to May 2022. The SEIEL, a 16-item validated instrument specifically designed to assess self-efficacy beliefs in interprofessional learning among students in diverse health professions,[20] was derived from the Interprofessional Collaborative Competencies Attainment Survey, the gold-standard tool for measuring competencies related to IPE.[21] Each item on the SEIEL was scored using a 10-point Likert-type scale (1 = *low confidence,* 10 = *very confident*). The SEIEL comprised two domains: (1) Interprofessional Interaction and (2) Interprofessional Team Evaluation and Feedback. The Interprofessional Interaction domain queried the students' attitudes and beliefs about their ability to work with students from different professions to form a team, resolve problems, and apply the team's work to benefit patient care. Perceived capabilities of communication, learning, and understanding regarding each team member's respective role within the team were ascertained in this domain. The Interprofessional Team Evaluation and Feedback domain assessed the students' understanding of the objectives of the interdisciplinary learning experience, as well as the students' perceived capability to evaluate themselves as a member of the team in order to determine if these learning objectives had been met. The students' level of confidence to provide feedback to the overall team and individual team members' performance were analyzed in this domain. Each domain could be scored independently, and the scores of the two domains could be scored together to derive a total score. The SEIEL possessed strong psychometric properties (Cronbach $\alpha =$ .96 for the total scale and .94 and .93 for the Interprofessional Interaction and Interprofessional Team Evaluation and Feedback domains, respectively).[20] Qualitative data were collected as part of the survey to provide additional commentary and identify the strengths and weaknesses of the activity.

Data Analysis

To determine if there were significant pre/post changes in self-efficacy for interprofessional learning, we analyzed the data via paired *t* tests for SEIEL total scores and the Interprofessional Interaction and Interprofessional Team Evaluation and Feedback domains. To reduce the possibility of Type I errors, a Bonferroni correction was utilized, and the level of significance was set at .017 (.05 divided by 3). Data were analyzed via SPSS Statistics for Windows, version 25 (SPSS Inc.). Qualitative data were obtained from each participant in written format via two open-ended question items posed at the end of the SEIEL survey: "Please identify the strengths of this IPE activity" and "Please identify areas that need improvement in this IPE activity." Written responses to the open-ended questions were analyzed independently by two investigators, and thematic analysis was performed.

Results

A total of 82 students participated in the IPE activity. Twenty-two PT and 53 medical students completed the SEIEL questionnaire

Copyright © 2023 Kung et al. This is an open-access publication distributed under the terms of the Creative Commons Attribution license.

before and after the IPE activity (response rate = 91%). The remaining seven students, who were not included in the data analysis, either had missing data or did not complete the survey. There were significant improvements in self-efficacy to engage in interprofessional learning as evidenced by higher scores on the SEIEL total and two domain scores (Table). The mean change in the SEIEL total score before and after the intervention was 32.0 ($p < .001$). The mean changes in the Interprofessional Interaction and Interprofessional Team Evaluation and Feedback domains before and after the intervention were 15.4 and 16.6, respectively ($p < .001$).

Qualitative comments indicated that the activity was an eye-opening experience to "see what the other profession did" and that it was "gratifying to be able to collaborate" to help the patients. Students noted that after the IPE activity, they had a greater appreciation for the other discipline. Many students recognized that their exam skills expanded after working with the other discipline and also that their ability to ask questions and communicate with the other discipline improved. Students felt that the interaction with the other discipline increased their confidence to consult with each other. A representative comment expressed that the student wished the activity had occurred earlier in their medical school career so that they could "use this knowledge to provide better patient care throughout [their] rotations."

Discussion

This IPE activity was incorporated into the workplace for medical students while on their neurology clerkship and PT students training at our county hospital. Overall, students appreciated the opportunity to work together in the hospital with live patient interaction. This IPE activity gave them a broader perspective of the unique interplay between PT and medicine. Our IPE activity is distinct for its contribution to remedying the paucity of available literature on integrated workplace IPE activities in neurology clerkships with medical students and physical therapists.

In reference to social cognitive theory, the concept of self-efficacy is that ultimately different influences affect humans' motivation and influence future action.[14] We have demonstrated that all our learners exhibited significant change in their confidence and capability to understand and explain the importance of interprofessional communication and their individual roles and responsibilities on an interprofessional collaborative team. Qualitative data suggested that learners were particularly impressed by the other profession's approach to patient care and that the experience would help them in the future. Specifically, we have established that this IPE activity meets the objectives of improving students' skills in describing their own unique contributions to the medical team and demonstrating effective communication using verbal and written methods. Response rates were high, showing that the IPE activity was well received by the participants. The IPE activity let students show off what they knew about their patient's neurological disorder from their professional perspective and allowed them to clearly communicate their roles and responsibilities to others. Lastly, the students could take these complementary skills and apply them to patient care. Qualitative comments suggested that students gained greater self-confidence (or improvement in self-efficacy) in interprofessional interactions, increasing the likelihood of future collaboration.

The advantage of this IPE activity is that it provided an authentic and relevant clinical patient experience in a neurology curriculum for medical and PT students. The IPE activity allowed students to focus on each other's profession and offered a forum for discussion in small groups. All our learners felt it was helpful to observe the other profession examine the patient and discuss their approach to patient care. Students enhanced their communication skills via verbal and written methods and were provided with real-time feedback on their communication. Medical students felt they better understood the goals of therapy and its relation to disposition planning. PT students felt they gained an understanding of the patient from a medical

Table. Pre/Post SEIEL: Total and Domain Scores ($N = 75$)

Domain	*M*	*SD*	*M* Difference (*SD*)	p^a
Total SEIEL				
Before	111.2	20.5		
After	143.2	12.7	32.0 (18.7)	<.001
Interprofessional Interaction				
Before	57.7	10.9		
After	73.1	6.2	15.4 (9.2)	<.001
Interprofessional Team Evaluation and Feedback				
Before	53.5	10.5		
After	70.1	7.1	16.6 (10.1)	<.001

Abbreviation: SEIEL, Self-Efficacy for Interprofessional Experiential Learning.
[a]Paired *t* tests, level of significance set at .017 (.05 divided by 3).

Copyright © 2023 Kung et al. This is an open-access publication distributed under the terms of the Creative Commons Attribution license.

perspective. Since the patients were live patients admitted to the hospital, medical and therapy updates were very pertinent to their current state and medical/therapeutic care. In many cases, this enhanced patient care and disposition planning. The patients also benefited from hearing how the medical team participated in their care and shared collaborative ideas during the bedside rounds. Finally, this activity not only fulfilled an accreditation requirement for the medical and PT schools but also served as an attractive recruiting option for PT students and residents applying for these PT programs. At our institution, we are truly fortunate to have knowledgeable PT residents who facilitate the regularly scheduled activities. However, this activity could also be facilitated by PT faculty or neurology or physical medicine and rehabilitation faculty or residents.

Another advantage of this teaching activity was its integration into the neurology clerkship, where it did not require additional funding sources. As noted in our review of the literature, other IPE activities that focus on communication, roles, and responsibility objectives are often taught as simulated experiences. Simulation can be expensive, particularly when standardized patients are used. For that reason, most simulation experiences teach low-frequency but high-impact clinical skills. We posit that our IPE activity is excellent for foundational knowledge and skills associated with interprofessional collaboration in a complex environment.

Challenges for this IPE activity included scheduling of the sessions to coincide with medical and PT student academic schedules, logistical constraints during the COVID-19 pandemic, and ensuring alignment with both the medical and PT students' curricula. One of the lessons learned was that occasionally some of the inpatients were unavailable or had been discharged prior to the day of the IPE activity. We controlled for this potential occurrence by having the facilitator review the patient list the day prior to the session and identify an alternative patient if needed. Another lesson learned was that, as health care providers, physical therapists and medical students alike often wanted to discuss the medical and therapeutic aspects of the patient's care. Although this was clinically relevant, we had to be vigilant to not lose sight of the IPE objectives. Outlining the goals of the IPE activity through a brief orientation prior to each session was effective for ensuring that the objectives were clear. The facilitator often being an active practitioner in the patient's care was essential for directing the conversation towards these objectives while still providing pertinent medical/therapy perspectives.

One of the limitations of this project is that its results may not be generalizable to other clerkships or other medical specialties since our IPE activity is focused on neurological disorders. Limitations for implementation at other institutions include number of patients available to participate, facilitators knowledgeable in neurologic patient care, and scheduling conflicts between facilitators, PT students, and medical students. Time commitment is initially greater to orient and train facilitators, but once trained, facilitators usually spend about 5 hours per session—1 hour for preparation and 4 hours for the IPE session (Figure). Having a lead faculty or coordinator send email reminders prior to the activity every month is valuable for maintaining the IPE activity. Annually, it is helpful to align the curricular schedules of the PT and medical students so that the activity is already planned at the beginning of the school year.

In subsequent iterations of this IPE activity, we plan to measure implications of the intervention on student behavior and patient outcomes. Other considerations include incorporating neurology residents and other professions into the activity as well. IPE activities like this should be encouraged and developed to reach more students in a neurology curriculum and can be applied broadly and modified for other interactions between medicine and therapy.

Appendices

A. Facilitator Guide.docx
B. IPE on the Neurology Clerkship.pptx
C. Example Schedule.docx
D. SEIEL Survey.docx
E. Neurological Medical Exam Example.docx
F. PT Neurological Exam Example.docx

All appendices are peer reviewed as integral parts of the Original Publication.

Doris Kung, DO: Associate Professor, Department of Neurology, and Assistant Dean of Clinical Curriculum, Baylor College of Medicine

Wayne Brewer, PT, PhD, MPH: Associate Professor, School of Physical Therapy, Texas Woman's University

Victor Oyelami, DPT: Doctor of Physical Therapy, MD Anderson Cancer Center, University of Texas

Stephanie Hessel, DPT: Doctor of Physical Therapy, Harris Health System

Laurene Bramlett, DPT: Doctor of Physical Therapy, Harris Health System

Copyright © 2023 Kung et al. This is an open-access publication distributed under the terms of the Creative Commons Attribution license.

Anne Gill, DrPH: Professor, Department of Pediatrics and Center for Medical Ethics and Health Policy, and Assistant Dean of Interprofessional Education, Baylor College of Medicine

Disclosures
None to report.

Funding/Support
None to report.

Prior Presentations

Kung DH, Oyelami V, Brewer W, Hessel S, Bramlett L, Gill A. Interprofessional education on the neurology clerkship involving physical therapy and medical students. Poster presented virtually at: Learn Serve Lead 2021: the AAMC Virtual Conference, November 8-10, 2021.

Kung DH, Oyelami V, Brewer W, Hessel S, Bramlett L, Gill A. Interprofessional education on the neurology clerkship involving physical therapy and medical students. Poster presented at: Baylor College of Medicine Annual Showcase of Educational Scholarship; September 16, 2021; Houston, TX.

Kung DH, Oyelami V, Brewer W, Hessel S, Bramlett L, Gill A. Interprofessional education on the neurology clerkship involving physical therapy and medical students. Poster presented virtually at: TEACH-S Educational Symposium; May 14, 2021.

Ethical Approval

The Baylor College of Medicine Institutional Review Board approved this project, and the Harris Health System Institutional Review Board deemed further review of the project not necessary.

References

1. GBD 2016 Neurology Collaborators. Global, regional, and national burden of neurological disorders, 1990–2016: a systematic analysis for the Global Burden of Disease Study 2016. *Lancet Neurol*. 2019;18(5):459-480. https://doi.org/10.1016/S1474-4422(18)30499-X
2. Cohen EV, Hagestuen R, González-Ramos G, et al. Interprofessional education increases knowledge, promotes team building, and changes practice in the care of Parkinson's disease. *Parkinsonism Relat Disord*. 2016;22:21-27. https://doi.org/10.1016/j.parkreldis.2015.11.001
3. Bahouth MN, Raghavan P, Tenberg A, et al. Abstract P171: JSTTEP: an interprofessional intervention to reduce early risk after hospitalization for stroke. *Stroke*. 2021;52(suppl 1):AP171. https://doi.org/10.1161/str.52.suppl_1.P171
4. Interprofessional Education Collaborative. *Core Competencies for Interprofessional Collaborative Practice: 2016 Update*. Interprofessional Education Collaborative; 2016. Accessed March 13, 2023. https://ipec.memberclicks.net/assets/2016-Update.pdf
5. Liaison Committee on Medical Education. *Functions and Structure of a Medical School: Standards for Accreditation of Medical Education Programs Leading to the MD Degree*. Liaison Committee on Medical Education; 2022. Accessed March 13, 2023. https://lcme.org/wp-content/uploads/2022/07/2023-24_Functions-and-Structure_2022-03-31.docx
6. Kent F, Nankervis K, Johnson C, Hodgkinson M, Baulch J, Haines T. "More effort and more time": considerations in the establishment of interprofessional education programs in the workplace. *J Interprof Care*. 2018;32(1):89-94. https://doi.org/10.1080/13561820.2017.1381076
7. Irfan M, Tariq N, Hussein H, Fitzgerald-Swenson L, Hart D, Tiryaki E. Stroke code simulation for medical education. *MedEdPORTAL*. 2015;11:10009. https://doi.org/10.15766/mep_2374-8265.10009
8. Karpa K, Pinto C, Possanza A, et al. Stroke simulation activity: a standardized patient case for interprofessional student learning. *MedEdPORTAL*. 2018;14:10698. https://doi.org/10.15766/mep_2374-8265.10698
9. Sizemore JN, Kurowski-Burt A, Evans K, Hoffman A, Summers A, Baugh GM. Interdisciplinary education apartment simulation (IDEAS) project: an interdisciplinary simulation for transitional home care. *MedEdPORTAL*. 2021;17:11111. https://doi.org/10.15766/mep_2374-8265.11111
10. Arth KS, Shumaker EA, Bergman AC, Nolan AM, Ritzline PD, Paz JC. Physical therapist student outcomes of interprofessional education in professional (entry-level) physical therapist education programs: a systematic review. *J Phys Ther Educ*. 2018;32(3):226-240. https://doi.org/10.1097/JTE.0000000000000059
11. Melrose S, Park C, Perry B. *Creative Clinical Teaching in the Health Professions*. AU Press; 2021.
12. Carey MP, Forsyth AD. Teaching tip sheet: self-efficacy. American Psychological Association. 2009. Accessed March 13, 2023. https://www.apa.org/pi/aids/resources/education/self-efficacy
13. Nørgaard B, Draborg E, Vestergaard E, Odgaard E, Jensen DC, Sørensen J. Interprofessional clinical training improves self-efficacy of health care students. *Med Teach*. 2013;35(6):e1235-e1242. https://doi.org/10.3109/0142159X.2012.746452
14. Davidson J, Davidson F. *Bandura's Social Cognitive Theory: An Introduction*. Davidson Films; 2003.
15. Egenberg S, Øian P, Eggebø TM, Arsenovic MG, Bru LE. Changes in self-efficacy, collective efficacy and patient outcome following interprofessional simulation training on postpartum haemorrhage. *J Clin Nurs*. 2017;26(19-20):3174-3187. https://doi.org/10.1111/jocn.13666
16. Holliday L, Carter T, Reddy H, Clarke L, Pearson M, Felton A. Shared learning to improve the care for young people and mental health within nurse education (SHYNE): improving attitudes, confidence and self-efficacy. *Nurse Educ Pract*. 2020;46:102793. https://doi.org/10.1016/j.nepr.2020.102793

Copyright © 2023 Kung et al. This is an open-access publication distributed under the terms of the Creative Commons Attribution license.

17. Hough J, Levan D, Steele M, Kelly K, Dalton M. Simulation-based education improves student self-efficacy in physiotherapy assessment and management of paediatric patients. *BMC Med Educ*. 2019;19:463. https://doi.org/10.1186/s12909-019-1894-2

18. Milam LA, Cohen GL, Mueller C, Salles A. The relationship between self-efficacy and well-being among surgical residents. *J Surg Educ*. 2019;76(2):321-328. https://doi.org/10.1016/j.jsurg.2018.07.028

19. Sharour LA. Improving oncology nurses' knowledge, self-confidence, and self-efficacy in nutritional assessment and counseling for patients with cancer: a quasi-experimental design. *Nutrition*. 2019;62:131-134. https://doi.org/10.1016/j.nut.2018.12.004

20. Mann K, McFetridge-Durdle J, Breau L, et al. Development of a scale to measure health professions students' self-efficacy beliefs in interprofessional learning. *J Interprof Care*. 2012;26(2):92-99. https://doi.org/10.3109/13561820.2011.640759

21. Schmitz CC, Radosevich DM, Jardine P, MacDonald CJ, Trumpower D, Archibald D. The Interprofessional Collaborative Competency Attainment Survey (ICCAS): a replication validation study. *J Interprof Care*. 2017;31(1):28-34. https://doi.org/10.1080/13561820.2016.1233096

Received: August 29, 2022
Accepted: February 2, 2023
Published: May 30, 2023

Copyright © 2023 Kung et al. This is an open-access publication distributed under the terms of the Creative Commons Attribution license.
